# Preliminary study of canine distemper virus transmission from small mammals to Malayan tiger at Kampung Besul Lama, Terengganu, Malaysia

**DOI:** 10.14202/vetworld.2025.791-798

**Published:** 2025-04-07

**Authors:** Bryan Andrew Lazarus, Muhammad Farris Mohd Sadali, Farina Mustaffa Kamal, Khor Kuan Hua, Ridhwan Abdul Wahab, Mohd Arifin Kaderi, Mohd Lutfi Abdullah, Tengku Rinalfi Putra Tengku Azizan, Hafandi Ahmad

**Affiliations:** 1 Department of Veterinary Preclinical Sciences, Faculty of Veterinary Medicine, Universiti Putra Malaysia, 43400, UPM Serdang, Selangor, Malaysia; 2 Department of Veterinary Pathology and Microbiology, Faculty of Veterinary Medicine, Universiti Putra Malaysia, 43400, UPM Serdang, Selangor, Malaysia; 3 Department of Clinical Studies, Faculty of Veterinary Medicine, Universiti Putra Malaysia, 43400, UPM Serdang, Selangor, Malaysia; 4 Faculty of Health Sciences, University College of Malaysian Federal Territory Islamic Religious Council (MAIWP) International, Taman Batu Muda, 68100 Batu Caves, Kuala Lumpur, Malaysia; 5 Department of Biomedical Science, Kulliyyah of Allied Health Sciences, International Islamic University Malaysia, Kuantan Campus, Jalan Sultan Ahmad Shah, 25200 Kuantan, Pahang; 6 National Wildlife Forensic Laboratory, ex situ Conservation Division, Department of Wildlife and National Parks Peninsular Malaysia, Kuala Lumpur, Malaysia

**Keywords:** canine distemper virus, Malayan tiger, molecular detection, small mammals, wildlife conservation, zoonotic spillover

## Abstract

**Background and Aim:**

The increasing human-wildlife interface due to urbanization and agricultural expansion has escalated the risk of zoonotic and interspecies disease transmission. Canine distemper virus (CDV), a highly contagious *Morbillivirus*, has been documented in various carnivorous and non-carnivorous species. In 2019, Malaysia reported its first case of CDV infection in a wild Malayan tiger (*Panthera tigris malayensis*) named Awang Besul in Kampung Besul Lama, Terengganu. However, the potential role of small mammals as intermediate hosts in CDV transmission remains poorly understood. This study aimed to investigate the role of small mammals as potential reservoir hosts for CDV and to provide molecular confirmation of CDV infection in these species, thereby assessing their role in enzootic viral maintenance and cross-species transmission to apex predators like the Malayan tiger.

**Materials and Methods:**

Wildlife sampling was conducted between July 2023 and May 2024 in Kampung Besul Lama, where CDV was previously detected in a Malayan tiger. A total of 77 small mammals from different species were captured using baited live traps. Species identification was performed based on morphological characteristics. Biological samples were collected through nasal and ocular swabs and analyzed using reverse transcription polymerase chain reaction to detect CDV RNA. Positive isolates were subjected to sequencing and Nucleotide Basic Local Alignment Search Tool analysis for molecular characterization.

**Results:**

Molecular detection confirmed CDV RNA in three common tree shrews (*Tupaia glis*), marking the first documented case of CDV in this species. Phylogenetic analysis of the viral hemagglutinin (*H*) gene revealed a 99.50% nucleotide similarity to a previously reported Malaysian CDV strain (BesulMY/Malaysia/PP894823.1). These findings suggest that small mammals may act as overlooked reservoir hosts, facilitating viral maintenance and spillover between domestic animals and wildlife.

**Conclusion:**

This study provides the first molecular evidence of CDV infection in tree shrews, highlighting their potential role in sustaining CDV in an enzootic state and acting as a conduit for interspecies transmission. Given the critically endangered status of Malayan tigers, targeted CDV surveillance and One Health-based disease mitigation strategies are essential to prevent further spillover events that could accelerate species decline.

## INTRODUCTION

The population and home range of Malayan tigers (*Panthera tigris malayensis*) have declined steadily to <150 individuals in the latest National Tiger Survey [1]. In fact, a decline in the population has been attributed to habitat loss due to deforestation and plantation, poaching for the illegal trade of tiger parts, killings in areas of human-tiger conflict, as well as hunting of the tiger’s natural prey population [2]. Urbanization and agricultural expansion have decimated forests in Malaysia with an annual estimate of 0.43 million ha/y, leading to habitat fragmentation, constriction of species movement, and human encroachment into wild territories [3, 4]. Domestic animal diseases such as canine distemper [5], feline parvovirus [6], and feline immunodeficiency virus [7] have been reported in wild tigers, suggestive of disease transmission from domestic groups to wild species. Canine distemper virus (CDV) of the genus *Morbilivirus* and family *Paramyxoviridae* is a single-strand RNA virus infecting carnivorous mammals and causes a highly contagious infection with a high mortality rate [8, 9]. CDV has been reported to infect a wide range of carnivorous hosts: Dogs, ferrets, wild dogs, foxes, jackals, coyotes, hyenas, cheetahs, lions, leopards, and tigers [10]; however, infections in non-carnivores have also been documented, such as in *Primates* (Japanese macaques, Rhesus macaques, and Squirrel monkey, *Xenarthra*’*s* (Giant anteater, Linnaeus’s 2-toed sloth, Southern tamandua), *Tayassuidae* (Collared peccary), *Pholidota* (Formosan pangolin), and Rodentia (Asian marmot) [11]. The transmission of the virus to various hosts includes direct contact, oral, ocular, and respiratory discharges, as well as viral toxins [12]. The pathophysiological effects of CDV include multiple organ failure, respiratory and gastrointestinal signs, secondary bacterial infection, neurological symptoms, and severe transitory immunosuppression [10].

The increasing human-wildlife interface not only intensifies habitat fragmentation but also introduces a new paradigm in disease ecology, whereby small mammals may sustain pathogen transmission cycles, posing unforeseen conservation threats. While previous research has established CDV transmission in large carnivores, this study is the first to examine small mammals as potential viral reservoirs, emphasizing their role in maintaining CDV in an enzootic state and facilitating indirect disease transmission to Malayan tigers. A previous study by Gilbert [13] has attributed CDV spillover to domestic animal predation; however, our study introduces an alternative hypothesis: Small mammals such as civets and tree shrews may function as intermediary hosts, maintaining CDV circulation between human settlements and forested ecosystems. The potential for smaller mammals such as civets and tree shrews to harbor and transfer diseases must be acknowledged, considering their movement in and out of human settlements in search of food. In addition, smaller mammals exist in much larger populations, enabling viruses such as the CDV to propagate and maintain an enzootic state [14]. This study also aligns with the One Health framework, demonstrating that wildlife conservation must incorporate disease surveillance at the domestic wildlife interface to mitigate emerging infectious diseases.

In Malaysia, a case of CDV has been reported infecting a wild Malayan Tiger in *Awang Besul* in Kampung Besul Lama, Terengganu. This headline case attracted national controversy because a timid tiger roamed around a village and displayed no signs of aggression. Although *Awang Besul* is the first reported case of CDV in Malayan tigers in Malaysia, this novel finding may indicate a more widespread threat to critically endangered Malayan tigers.

Despite extensive research on CDV transmission in large carnivores, the role of small mammals as potential reservoir hosts in the enzootic maintenance of CDV remains underexplored. Previous studies have primarily focused on direct transmission from domestic animals to wildlife, neglecting the possibility of an indirect transmission cycle involving small mammal populations. Given the first confirmed CDV infection in a Malayan tiger *(Awang Besul*) in Malaysia, it is imperative to investigate alternative transmission pathways, particularly through small mammals that interact with both domestic and wild species. Understanding these overlooked reservoirs is critical for developing effective wildlife disease surveillance and conservation strategies.

This study aims to identify and characterize potential reservoir hosts of CDV among small mammals in Kampung Besul Lama, Terengganu, where CDV was previously detected in a wild Malayan tiger. Specifically, the study seeks to molecularly confirm CDV presence in small mammals, assess their role in cross-species transmission, and provide insights into the enzootic maintenance of the virus. Findings from this study will contribute to improving wildlife disease management strategies and conservation efforts for the critically endangered Malayan tiger.

## MATERIALS AND METHODS

### Ethical approval

The permit for wildlife research at the Kampung Besul Lama was approved by the Department of Wildlife National Parks (PERHILITAN), Peninsular Malaysia, Reference: 100–34/1.24 Jld20 (11). All animal handlings were conducted according to the principles of the Laboratory Animal Care and Use Committee (Institutional Animal Care and Use Committee) approved by the Universiti Putra Malaysia (UPM/ IACUC/AUP-R092/2017).

### Study period and location

**Figure 1:**
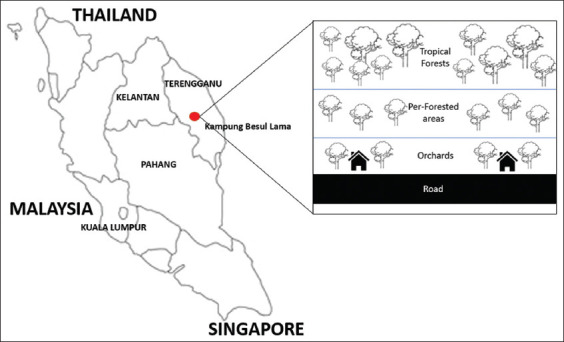
Location of Kampung Besul Lama, Malaysia [Source: https://openclipart.org/image/400px/222321].

The study was conducted from 19^th^ July 2023 to 10^th^ May 2024 at the Kampung Besul Lama, Terengganu. This area was chosen as the first study location due to the confirmed presence of the CDV in a Malayan tiger. Figure 1 shows the location of Kampung Besul Lama, which is connected to the forested area, orchards, and village settlements. The orchard areas at Kampung Besul Lama were chosen as study sites due to the high changes in a mixture of domestic species as well as the presence of wild small mammals.

### Wildlife trapping and sampling using baited traps

**Figure 2:**
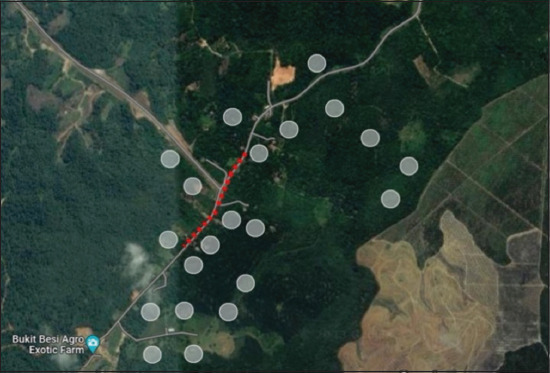
Location of the areas in which traps were placed during wildlife sampling (gray circle). The movement of *Awang Besul* is shown on a map (red line) [Source: https://earth.google.com/earth/d/1A_VYXtrya9lqPdqdaLWfghu-hmgeT7qN?usp=sharin].

Wildlife traps of two sizes were used for small mammals. First set of traps (n = 10; 18 cm × 29 cm × 13 cm) was aimed at smaller mammals, such as squirrels or rats. The second set of traps (n = 10; 40 cm × 30 cm × 65 cm) targeted larger mammals such as civets, wild cats, and other possible mammals. Both traps were spring-live cage traps made of steel with a locking mechanism to prevent escape. The traps contain a spring mechanism at the back end that causes the trap to close shut when triggered and a lock mechanism that prevents the animal from escaping by locking the door shut. These wildlife traps were placed in peri-forested and orchard areas, as well as in areas of movement where *Awang Besul* was detected. Traps aimed at smaller mammals were placed on trees, nearby food sources (e.g. fruit trees), and on the ground in bushes. Traps aimed at larger mammals were placed in bushes, along wildlife pathways, and around village areas where chicken coops were present. The traps were baited with bananas, jackfruit, and palm oil kernels. The traps were checked twice daily (morning and evening) and the baits were replaced if damaged or spoilt. Figure 2 shows the locations of the areas in which traps were placed during wildlife sampling (gray circle) and the movement of *Awang Besul* (red line).

Captured animals were anesthetized following species-specific protocols [15–17], ensuring ethical handling in accordance with established wildlife veterinary guidelines. Animal species were identified according to the field guide for the mammals of Southeast Asia [18]. Nasal and oral samples were collected using sterile swabs and preserved in a virus transport medium (BMS Diagnostics, Malaysia) for the detection of CDV through RT-PCR. To maintain RNA integrity, samples were transported on dry ice and immediately processed upon arrival at the laboratory. Future studies should incorporate metagenomic sequencing to analyze viral evolution and use GPS tracking to validate small animal movement patterns that may facilitate disease spillover. Anesthetized mammals were placed in a dark area for recovery and released after 24 h into the same site where they were captured.

### RT-PCR

Viral RNA was extracted using the Nucleospin RNA virus protocol (Macherey-Nagel, Germany). Conversion to cDNA was done using a SensiFAST^™^ cDNA synthesis kit (Bioline, United Kingdom) according to the manufacturer’s protocol. The cDNA templated were subjected to PCR amplification using a primer pair (Forward: 5’-TTCATCCAAGCTGTCCTTAGTG-3’; Reverse: 5’-GTGATGTACGGCCTCTGATTT-3’) amplifying an amplicon of 202 bp of the viral hemagglutinin (*H*) gene for qualitative determination of the presence of CDV RNA. PCR was done using MyTaq^™^ RedMix (Bioline, United Kingdom), following the manufacturer’s protocol. The PCR cycle was as follows: Initial denaturation at 95°C for 1 min; 30 cycles of denaturation at 95°C for 1 min; annealing at 54.7°C for 1 min; extension at 72 °C for 50 s and a final extension at 72°C for 3 min. The PCR product was visualized through 1.5% agarose gel electrophoresis in 1× Tris-acetate-ethylenediaminetetraacetic acid buffer (Bio-Rad, USA) and stained with 1 μL of Redsafe^™^ solution (iNtRON Biotechnology, South Korea) against the Recombitek C3 vaccine (Boehringer Ingelheim, Germany) and nuclease-free water as positive and negative controls, respectively. The gel was examined under an ultraviolet illuminator (Syngene, USA).

To confirm that the PCR product was CDV RNA, the PCR amplicons of positive isolates were purified and sequenced using an ABI PRISM 3,730 × l Genetic Analyzer (Applied Biosystems, USA) with the primer set described in this study. The Nucleotide Basic Local Alignment Search Tool (BLASTn) was used to compare the sequence similarity of the partial *H* gene sequence of the positive isolates with all the CDV sequences in NCBI GenBank. This study represents the first known RT-PCR detection of CDV in small mammals in Malaysia, marking a critical step in understanding how viral reservoirs contribute to disease persistence in wildlife populations.

## RESULTS AND DISCUSSION

This study provides the first molecular evidence of CDV infection in tree shrew *Tupaia glis*, supporting the hypothesis that small mammals may act as reservoir hosts facilitating disease transmission between domestic and wild species. Unlike previous research that has focused on direct domestic wildlife transmission, our findings suggest an alternative enzootic maintenance cycle within small mammal populations. Table 1 lists the species of small mammals captured and recorded. Sampling yielded a total of 77 small mammals: *T. glis* (n = 16), *Callosciurus notatus* (n = 9), *Callosciurus caniceps* (n = 7), *Sundasciurus tenuis* (n = 9), *Paradoxurus hermaphroditus* (n = 8), *Viverra tangalunga* (n = 1), *Leopoldamys sabanus* (n = 1), and *Rattus rattus* (n = 26). These small mammals are believed to be potential causative agents of the spillover of the CDV from domestic areas to wildlife habitats. In addition to their ability to be infected by the virus, these small mammals exist in large numbers, enabling the maintenance of the CDV within their population in an enzootic state. Social interactions are common between species, such as tree shrews, and facilitate virus transmission and maintenance. Previous studies by [10, 11] on CDV have shown that its potential hosts are extensive. The virus has been detected in a wide range of species, including domestic dogs, ferrets, wild canids (such as foxes, jackals, and coyotes), hyenas, cheetahs, and large felids such as lions, leopards, and tigers [10]. In addition, CDV has been identified in some non-carnivorous species across other orders, such as *Primates*, *Xenarthra*, *Pholidota*, and Rodentia [11].

**Table 1:** Captured small mammals and detected antigen-positive individuals.

Species	Number of individuals (n)	Relative abundance (n/N×100)	CDV antigen positivity
Common tree shrews *Tupaia glis*	16	20	3
Plantain squirrel *Callosciurus notatus*	9	11	0
Gray-bellied squirrel *Callosciurus caniceps*	7	9	0
Slender squirrel *Sundasciurus tenuis*	9	11	0
Asian palm civet *Paradoxurus hermaphroditus*	8	10	0
Malayan civet *Viverra tangalunga*	1	1	0
Long-tailed giant rat *Leopoldamys sabanus*	1	1	0
Field rat *Rattus rattus*	26	33	0
Total	77		3

CDV=Canine distemper virus, n=numberof individuals of same species, N=number of individuals of all species

**Figure 3:**
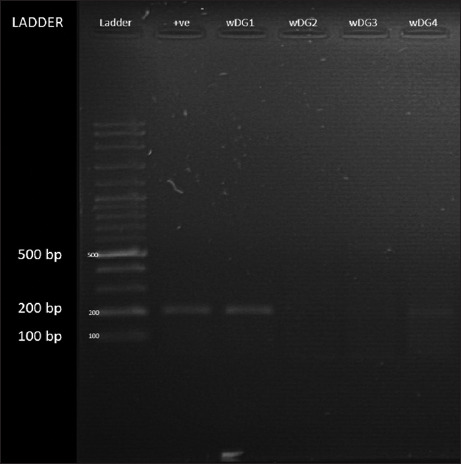
Qualitative identification through conventional reverse transcription polymerase chain reaction depicting results for tree shrew *Tupaia glis* (wDG1 and wDG4) samples positive for canine distemper virus RNA from Kampung Besul Lama, Terengganu based on selected region in the *H* gene.

Figure 3 shows the qualitative molecular identification through RT-PCR depicting antigen-positive results for tree shrew (*T. glis*) samples from Kampung Besul Lama based on a selected region in the *H* gene. RT-PCR confirmed the CDV antigen presence in three tree shrews *T. glis*, marking the first molecular detection of CDV in this species. BLASTn analysis revealed that the partial *H* gene sequences from the positive isolates exhibited high nucleotide identity with CDV sequences (Supplementary Figures). Sequence analysis revealed a 99.50% maximum nucleotide identity to the BesulMY/Malaysia/PP894823.1 CDV isolate, indicating a previously undocumented transmission pathway through small mammals. Nucleotide comparisons of PCR amplicons confirm that tree shrews are infected by CDV. Positive CDV detection in tree shrews suggests a high potential for spread to naive small mammals due to their large population size. Viral maintenance in reservoir populations has been demonstrated in other populations, such as the harp seal in the Northwest Atlantic [19]. The detection of CDV antigen in tree shrews suggests an unrecognized role of small mammals in sustaining viral transmission cycles. Given their high population densities and ecological overlap with domestic animals, tree shrews may serve as “silent spreaders,” necessitating urgent wildlife disease surveillance. We hypothesized that these positive hosts could be asymptomatic or in the recovery phase of the viral infection, thus not displaying symptoms but actively discharging the virus. The positive result obtained, and large population size indicates that the CDV is maintained in small mammal populations, enabling viral survivability and infectibility to naive species when the opportunity arises. The maintenance of CDV in small mammal populations has been demonstrated in Germany and the USA, where raccoons were responsible for the spread of the disease to domestic and wild species, with the virus being maintained in the raccoon population [20, 21]. This study suggests that tree shrews contribute to an enzootic maintenance cycle of CDV due to their high population densities and movement between human settlements and forested areas, acting as a disease reservoir and facilitating indirect transmission to Malayan tigers.

Results of PCR in qualitative analysis indicate that small mammals are the reservoir hosts for CDV transmission and are responsible for infection in wild mammals. Despite testing negative for other captured mammals, this does not indicate that they have never been exposed or recovered from a CDV infection. A previous study by Kim *et al*. [22] reported reduced sensitivity and specificity with RT-PCR, with dissatisfactory detection during the early and end stages of the infection. In addition, we hypothesize that these small mammals move between human settlements and forested areas for forage and, coincidentally, transmit disease. Mapping the movements of small mammals would enable us to predict the possibility of disease transference. Due to the connectivity of some forests in Malaysia, the maintenance of CDVs in small mammal populations could prove devastating to endangered species. Kampung Besul Lama in Terengganu is the first reported CDV in a wild Malayan tiger. The forest that hosts *Awang Besul* in Terengganu is also connected to the National Park in Pahang, Malaysia. If the transmission of CDV ever reaches the National Park, the remaining minuscule tiger population will be at severe risk of extinction.

**Figure 4:**
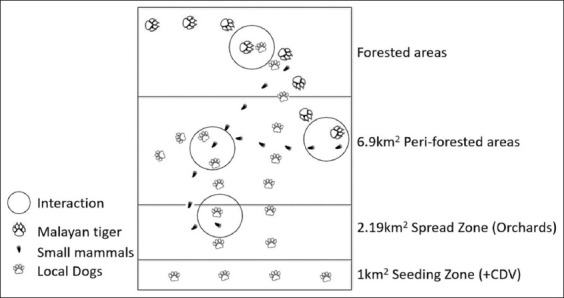
Hypothesis on interaction and transmission theory of canine distemper virus between village dogs, small mammals, and Malayan tiger. The distance was calculated using Google Earth.

The theory of transmission of the CDV to *Awang Besul* is depicted in Figure 4, showing certain areas of potential interaction. The origin of CDV infection in Kampung Besul Lama remains undetermined; however, the landscape of Kampung Besul Lama provides ample interactions between CDV-susceptible animals such as dogs and small mammals in the form of fruit orchards bordering peri-forested areas, palm oil plantations, and tropical forests. Sharing of the same environment between roaming dogs and small mammals creates an optimal environment for virus transmission, suggesting that CDV transmission to tree shrews can potentially maintain the virus in the population. Regarding *Awang Besul*, the fragmented forests surrounding the village areas may facilitate tiger movement through villages in search of food and other socioecological interactions. This could lead to possible predation of infected dogs or movement into regions inhabited by infected small mammals, thereby increasing the risk of infection and death of tigers. According to Gilbert *et al*. [7], pathogen emergence in wildlife may be attributed to modified habitats with reduced biodiversity, altered climates, and fragmented landscapes. This is seen in Kampung Besul Lama, where indiscriminate logging has led to fragmented forests, causing wildlife movement through human settlements. In addition, the African swine fever outbreak in wild boars in Malaysia has concurrently reduced the prey population and caused predator movement to areas of human settlements in search of food [23].

## CONCLUSION

This study provides the first molecular evidence of CDV infection in tree shrews (*T. glis*), highlighting their potential role as reservoir hosts in the transmission network between domestic animals and Malayan tigers. These findings redefine the epidemiology of CDV by demonstrating an enzootic maintenance cycle within small mammal populations, a previously overlooked transmission pathway. The study also contributes to wildlife disease ecology by documenting small mammal species present in peri-forested areas and their potential involvement in viral spillover to apex predators. The insights gained from this research will serve as a foundation for enhanced wildlife conservation strategies and facilitate a more detailed exploration of disease ecology and host-pathogen interactions.

A key strength of this study lies in its novel molecular detection of CDV in tree shrews, marking the first confirmed case of CDV infection in this species. This research pioneers the integration of small mammals into One Health disease surveillance, expanding the traditional view of CDV transmission beyond direct domestic-to-wildlife pathways. The study also employs rigorous RT-PCR and sequencing techniques, ensuring the reliability of molecular confirmation. In addition, the research aligns with conservation priorities, providing valuable data that could inform targeted disease monitoring and intervention strategies for Malayan tigers.

Despite its contributions, this study has certain limitations. The sample size, while sufficient to establish molecular evidence, remains limited in geographic scope, warranting further investigation across broader habitats. In addition, serological studies were not conducted to assess prior CDV exposure in the sampled small mammals, which could provide a more comprehensive understanding of their role in disease maintenance. The study also lacks longitudinal tracking of small mammals, which could help confirm their movement patterns and interactions with other species in the transmission network.

To build upon these findings, future research should expand the geographic range of small mammal sampling to determine whether CDV maintenance in reservoir species is widespread. Serological and immunological studies should be conducted to assess exposure history and viral shedding patterns in small mammals. Metagenomic sequencing could provide deeper insights into the genetic variability of circulating CDV strains and their evolutionary relationships. In addition, GPS tracking and ecological modeling should be incorporated to map movement patterns of small mammals and assess their role in facilitating viral spillover. Finally, targeted vaccination programs for domestic dogs in forest-edge communities should be explored to mitigate the risk of continued CDV transmission to endangered wildlife.

Given the enzootic potential of CDV in small mammals, conservation strategies should incorporate targeted disease surveillance and vaccination programs to curb cross-species transmission. The critically endangered status of Malayan tigers necessitates immediate intervention, as the persistence of viral reservoirs in small mammal populations could accelerate their population decline. Without strategic One Health-based approaches, ongoing CDV circulation in small mammal populations could exacerbate conservation challenges. This study underscores the necessity of integrating wildlife disease monitoring into broader conservation frameworks to ensure that domestic animal disease surveillance accounts for spillover risks to endangered species.

## DATA AVAILABILITY

The supplementary figures are available from the corresponding author upon reasonable request. The locations and animals were available and included in this study. Some confidential data are restricted to protect endangered wildlife in the study area. The data can be available from the corresponding author for researchers who meet the criteria for access to confidential data.

## AUTHORS’ CONTRIBUTIONS

HA, FMK, KKH, RAW, MAK, and TKPTA: Conceptualized and designed the study. MLA: Preparation of proposal and permit. BAL and MFMS: Involved in sample collection and RT-PCR. BAL: Drafted the manuscript. BAL and HA: Reviewed and revised the manuscript. All authors have read and approved the final manuscript.
